# A Novel Valued Tolerance Rough Set and Decision Rules Method for Indoor Positioning Using WiFi Fingerprinting

**DOI:** 10.3390/s22155709

**Published:** 2022-07-30

**Authors:** Ninh Duong-Bao, Jing He, Luong Nguyen Thi, Khanh Nguyen-Huu, Seon-Woo Lee

**Affiliations:** 1 College of Computer Science and Electronic Engineering, Hunan University, Changsha 410082, China; duongbaoninh@hnu.edu.cn (N.D.-B.); jhe@hnu.edu.cn (J.H.); 2 Faculty of Mathematics and Informatics, Dalat University, Dalat 66100, Vietnam; 3 Faculty of Information Technology, Dalat University, Dalat 66100, Vietnam; luongnt@dlu.edu.vn; 4 Department of Electronics and Telecommunications, Dalat University, Dalat 66100, Vietnam; 5 Division of Software, Hallym University, Chuncheon 24252, Korea; senu@hallym.ac.kr

**Keywords:** indoor positioning, WiFi fingerprinting, RSS, rough set, valued tolerance, decision rules

## Abstract

In recent years, due to the ubiquitous presence of WiFi access points in buildings, the WiFi fingerprinting method has become one of the most promising approaches for indoor positioning applications. However, the performance of this method is vulnerable to changes in indoor environments. To tackle this challenge, in this paper, we propose a novel WiFi fingerprinting method that uses the valued tolerance rough set theory–based classification method. In the offline phase, the conventional received signal strength (RSS) fingerprinting database is converted into a decision table. Then a new fingerprinting database with decision rules is constructed based on the decision table, which includes the credibility degrees and the support object set values for all decision rules. In the online phase, various classification levels are applied to find out the best match between the RSS values in the decision rules database and the measured RSS values at the unknown position. The experimental results compared the performance of the proposed method with those of the nearest-neighbor-based and the random statistical methods in two different test cases. The results show that the proposed method greatly outperforms the others in both cases, where it achieves high accuracy with 98.05% of right position classification, which is approximately 50.49% more accurate than the others. The mean positioning errors at wrong estimated positions for the two test cases are 1.71 m and 1.99 m, using the proposed method.

## 1. Introduction

Over the past ten years, location-based services have rapidly developed. In these services, positioning is one of the most indispensable approaches. For outdoor environments, the global navigation satellite system (GNSS) can provide very reasonable positioning results, with an average error of 3–5 m [1]. However, this system is limited outdoors since it cannot guarantee the same accuracy indoors, where the satellite signals are reflected or blocked by the walls of buildings. Thus, there is an extreme demand to develop indoor positioning systems (IPSs).

Currently, there are various technologies used to track the position of a mobile user indoors. These technologies can be classified into two groups: building independent and building dependent. The first group includes the IPSs that do not rely on the infrastructure of a building to determine the user’s position. Some examples for this group are dead reckoning [2] and image-based [3] technologies. On the other hand, the IPSs that relate to the building where they are operated belong to the second group. For this group, we can divide it into two subgroups: the dedicated infrastructure required and the building’s infrastructure utilized. The former subgroup is defined as the IPSs that need the unpopular infrastructure to be set up in a building. Radio frequency identification [4], acoustic signal [5], or visible light communication [6] are some examples belonging to this group. The latter subgroup utilizes the common infrastructure in the building to create the IPSs such as Bluetooth [7,8] and WiFi [9,10]. Among these technologies, WiFi-based positioning [11,12,13] has drawn tremendous attention due to the widespread of WiFi infrastructure in indoor environments such as office buildings, hospitals, malls, etc. Moreover, the high popularization of smart devices such as smartphones or tablets and the compatible implementation of these devices with WiFi systems also leads to the large deployment of this technology for IPS. To enhance the performance of the WiFi-based positioning system, there are hybrid systems that combine the result of the WiFi with other technologies, such as pedestrian dead reckoning [2,9], camera [14], or magnetic field [15].

There are two main approaches for WiFi-based positioning: geometric and fingerprinting [16]. For the former approach, some common conventional positioning methods are the time of arrival [17], time difference of arrival [18], angle of arrival [19], etc. The positioning accuracy of these methods depends on the line-of-sight condition, which is hard to keep indoors, where there are many physical obstacles, such as walls or doors. For the latter one, the WiFi fingerprinting method is another popular method due to its low cost and ease of implementation on smart devices. However, the reflections, the multipath interference, or the changes in the environmental conditions could greatly degrade the performance of this method. Therefore, achieving a reliable and highly accurate WiFi fingerprinting-based positioning method is a challenging problem.

The traditional WiFi fingerprinting method has two phases: the offline and the online phases. In the offline phase, the radio map is built by collecting the RSS values from the available APs at different reference points (RPs) in a target area. The unique set of RSS values at one RP is defined as a fingerprint of that position. In the online phase, by comparing the measured RSS values at one unknown position with the RSS values from the radio map using different matching methods, the user’s position can be estimated.

The conventional rough set theory was first proposed by Pawlak [20] as an extension of classical set theory to deal with vague information. This theory conceives that information and knowledge in information systems are expressed via indiscernibility relations (equivalence relations) between objects in the object set. The rough set theory is used for big data mining and knowledge reduction [21,22] and can be applied in various fields such as information systems [23] or real estate market analysis [24,25]. To give a more flexible way to the rough set theory to handle the indiscernibility relation, Stefanowski and Tsoukias [26] introduced the valued tolerance relation and proposed the valued tolerance rough set and decision rules method (VTRS–DR). This method is often applied as a support tool in decision-making systems. Rough set theory based on valued tolerance relation calculates the valued tolerance relation for all objects in the decision table, resulting in a valued tolerance relation matrix where the values belong to [0, 1]. This is the extension of the conventional rough set theory proposed by Pawlak.

When applied to WiFi fingerprinting-based indoor positioning, the VTRS–DR works not only in the offline phase but also in the online phase. In the offline phase, the conventional RSS fingerprinting database is converted into a decision table where the RSS values are classified into different decision classes which correspond to the predefined RPs. Each tolerance granule corresponding to each object (i.e., one row in the decision table) is calculated based on the valued tolerance relation matrix. After calculating the values of the membership degrees of objects belonging to lower (upper) approximations of each decision class, the credibility degrees of decision rules (i.e., objects in the decision table) are also calculated. Besides that, the support object set values of decision rules are defined by the tolerance granule of objects. A new fingerprinting database with decision rules is constructed from the decision table that includes the credibility degrees and the support object set values for all decision rules. In the online phase, the valued tolerance relation is calculated between the RSS values in the new database and the measured RSS values at an unknown position. The best decision class (i.e., one RP) is chosen among a set of RPs via the proposed method by comparing various components such as the valued tolerance relations, the credibility degrees, the support object set values, and the Euclidean distances; thus, the user’s position can be estimated. In our work, it means estimating the unknown position as the RP closest to that position.

To deal with the instability in RSS values from APs, in this paper, a novel VTRS–DR-based WiFi fingerprinting for IPS is proposed. The VTRS–DR method provides an efficient computational structure and helps to solve the indoor positioning problem with high accuracy. The main contributions are figured out as follows:

- The VTRS–DR method is applied in the offline phase of the WiFi fingerprinting method by creating a new fingerprinting database with decision rules that includes the credibility degrees and the support object set values for all rules.
- The VTRS–DR method is also applied in the online phase of the WiFi fingerprinting method by estimating the user’s position based on the fingerprinting decision rules database and the measured RSS value to determine the user’s position.
- For performance evaluation, the proposed method is compared with the nearest neighbor-based and the random statistical methods to prove its superior positioning accuracy and robustness.

The rest of this paper is organized as follows. Section 2 discusses the related works. Section 3 describes the basis of the VTRS–DR method. Section 4 introduces the proposed positioning method. Experimental results are displayed in Section 5, and the conclusion is given in Section 6.

## 2. Related Works

Generally, there are two categories of WiFi fingerprint-based matching algorithms: deterministic and probabilistic. The deterministic algorithm is very common due to its ease of implementation and its possibility to work well in real time. The nearest neighbor (NN), KNN, and weighted KNN (WKNN) [27,28] are some of the popular deterministic algorithms. To handle the fluctuation of WiFi RSS values, as well as to normalize them, Ninh et al. [10] introduced the random statistical method in the offline phase. The method helped to create a standardized fingerprinting database. In the online phase, to figure out the unknown position of a user, the authors applied the Mahalanobis distance as the matching algorithm to improve the positioning accuracy. The experiments were conducted in different setup conditions in an office room. As a result, the maximum positioning error was only 0.75 m. In Reference [29], instead of using the common Euclidean distance in NN-based algorithms, Duong-Bao et al. implemented five distance measures and compared the positioning results in different settings such as changing the number of setup APs or changing the grid spacing between two RPs. The experimental results showed that the Chi-Squared distance is the best measure with a mean error of 1.17 m in two test cases. Even though these deterministic algorithms could reduce computational complexity, they used only a single reference fingerprint for each RP (i.e., a set of mean RSS values from available AP) for finding the best match between the measured RSS values at an unknown position and the fingerprints in the database, and this could lead to big positioning errors due to the instability of the RSS values.

On the other hand, the probabilistic algorithm needs to know the probability distribution of the RSS values from available APs at every RPs. Even though this algorithm can provide good accuracy, it is more complex than the deterministic algorithm, and the positioning performance relies on the computation of likelihood functions. Some popular probabilistic algorithms are the Kalman filter [9], particle filter [30], and hidden Markov models [31]. Using two Kalman filters, Zhuang et al. [32] fused the positional information from the micro-electromechanical system (MEMS) sensors and WiFi fingerprinting to enhance the tracking accuracy. The MEMS sensors helped to reduce the searching space of WiFi fingerprinting by using an extended Kalman filter, and another Kalman filter was applied to smooth the positioning result of WiFi fingerprinting. From two test scenarios, the results showed that the proposed method improved the accuracy by 47% and 28% with mean errors of 2.2 m and 2.6 m, respectively. In Reference [33], Deng et al. also used the extended Kalman filter in the integrated system, which consisted of pedestrian dead reckoning, WiFi fingerprinting, and special positions in a building (i.e., doors, elevators, escalators, etc.) to reduce the positioning error. From the experiments, it was shown that the mean positioning error was only 1.22 m in a test area of 487.2 m^2^. The two above methods obtained good positioning results; however, they could not completely handle the variation of the RSS values, even though they used different sources (i.e., smartphone sensors and special positions in a building), which would make the systems become more complicated, to calibrate for estimated user’s position.

Currently, many latest works use the WiFi fingerprinting technique as the main tool to determine the user’s position with the help of machine learning techniques [34,35,36,37,38]. In Reference [37], based on the conventional WiFi fingerprinting, Song et al. proposed the CNNLoc which combined the stacked auto-encoder and convolutional neural network (CNN) to track the users’ positions in multi-buildings with multi-floors. The framework required a lot of data preprocessing in the training phase, such as sub-datasets division, rectangle areas creation, cell grids division, etc. As the result, the proposed method obtained a 96.03% accuracy of floor classification with a positioning error of 11.78 m. Moreover, using the deep learning approach, Qin et al. [38] combined the convolutional denoising autoencoder (CDAE) and CNN for the IPS. In the offline phase, the K-means algorithm was used to extract the set of features. During the online phase, the RSS values were put into the CDAE to extract the main features, and then the CNN was used to find out the user’s position. The experimental results showed that the mean positioning errors were 1.05 m and 12.4 m for two different datasets. These methods were vulnerable to the changes of RSS values, and they were required to be adjusted to work well with different buildings.

The RSS values collected even from the same AP can fluctuate much due to the changes of environmental conditions, such as the number of working people, the number of electrical devices, the user’s body, the period in a day, etc. The aforementioned works have not controlled the instability of the RSS values perfectly. Therefore, we propose a novel WiFi fingerprinting-based method, which utilizes the valued tolerance rough set and decision rules method to classify the user’s position among predefined reference points. To the best of our knowledge, applying the VTRS–DR method to solve the indoor positioning challenge has never been recorded before.

## 3. VTRS–DR Method

Say we have a set of objects, $U=\left\{u_{1},u_{2},\ldots ,u_{m}\right\}$ (m is the total number of the fingerprints of *RP*s), that can be characterized by a set of conditional attributes, $C=\left\{RSS_{1},RSS_{2},\ldots ,RSS_{n}\right\}$ (a set of n *RSS* values collected from n APs at one *RP*, n<m). If we denote a subset of objects in U by a decision attribute d∉C (with $d\in \left\{RP_{1},RP_{2},\ldots ,RP_{N}\right\}$), which is one *RP* in a set of N *RP*s, then we define the decision table DT=(U, C∪{d}). The decision attribute d partitions set U into N decision classes (N≤m) as $D_{l}$, l=1, 2, …, N, with each class being one *RP* in the fingerprinting database. Each decision class is a tolerance class. At the beginning of the VTRS–DR method, the relations between the objects and the attributes can be represented through the decision table, DT, which is given in Table 1. In this table, n conditional attributes (i.e., n *RSS* values from n APs) $C=\left\{RSS_{1},RSS_{2},\ldots ,RSS_{n}\right\}$ are given and each attribute $RSS_{j}$, j=1, 2, …, n, has its *RSS* values changing in the interval [–100, 0] (dBm). Here, each object (i.e., one row in Table 1) represents one fingerprint (i.e., a unique set of RSS values) of the given *RP*, which is a decision class. It is worth noting that, in the decision table, DT, we do not need to sort the *RP*s by their orders, and the number of appearing times of each decision class (i.e., each *RP*) can be different which depends on the number of RSS scanning times at each *RP*.

To explain the VTRS–DR method clearly, we are able to give some basic definitions.

**Definition** **1.** *Valued tolerance relation among the objects of set*U*built on a set of conditional attributes*A⊆C*is denoted by*$R_{A}$ (*i.e.,* $R_{A}:U\times U\to \left[0,\;1\right]$). $R_{A}$ *satisfies two properties: (a) reflexive,* $\forall u\in U,\;R_{A}\left(u,u\right)=1$*; and (b) symmetric,* $\forall u,v\in U,\;R_{A}\left(u,v\right)=R_{A}\left(v,u\right)$.

Definition 1 is used to determine whether the objects of set U satisfy the valued tolerance relation built on a set of conditional attributes, A⊆C. If a pair of objects has the valued tolerance relation, then these objects may belong to the same tolerance class.

**Definition** **2.** *The valued tolerance relation of each attribute* $RSS_{j}$*, in any two objects,*u,v∈U*, is denoted by* $R_{j}\left(u,v\right)$*and calculated as follows:*

(1) $$R_{j}\left(u,v\right)=\frac{max\left(0,\;min\left(RSS_{j}\left(u\right),\;RSS_{j}\left(v\right)\right)+k_{j}-max\left(RSS_{j}\left(u\right),\;RSS_{j}\left(v\right)\right)\right)}{k_{j}}$$

where $RSS_{j}\in A\subseteq C$, $k_{j}>0$ is the discrimination threshold value of the attribute $RSS_{j}$, j=1, 2, …, n. The discrimination threshold, k, is defined as the threshold of the similarity measure. The value $k_{j}$ is used to measure the similarity between two values of the attribute $RSS_{j}$. From Equation (1), $\forall u,v\in U:\;R_{A}\left(u,v\right)=1\Leftrightarrow RSS_{j}\left(u\right)=RSS_{j}\left(v\right)$, $R_{A}\left(u,v\right)=0\Leftrightarrow \left|RSS_{j}\left(u\right)-RSS_{j}\left(v\right)\right|\geq k_{j}$, and $0<R_{A}\left(u,v\right)<1\Leftrightarrow \left|RSS_{j}\left(u\right)-RSS_{j}\left(v\right)\right|<k_{j}$. The value $k_{j}$ determines whether the two objects are indiscernible (equivalent) with the respect to each attribute in rough set theory.

Definition 2 is used to calculate the valued tolerance relation between a pair of objects in U built on a conditional attribute, $RSS_{j}\in A\subseteq C$. This definition supports the calculation of the valued tolerance relation between two objects in Definition 3.

**Definition** **3.** *The valued tolerance relation between two objects,* u,v∈U*, on a set of conditional attributes,* A⊆C*, is denoted by* $R_{A}\left(u,v\right)$*and calculated as follows:*

(2) $$R_{A}\left(u,v\right)=\underset{RSS_{j}\;\in \;A}{min}\left(R_{j}\left(u,v\right)\right)$$

From Equation (2), $\forall u,v\in U:\;0\leq R_{A}\left(u,v\right)\leq 1$, and $R_{A}\left(u,v\right)$ satisfies two properties, (a) and (b), of the valued tolerance relation.

Definition 3 used is to determine the valued tolerance relation between a pair of objects in U built on a set of conditional attributes, A⊆C. If $R_{A}\left(u,v\right)=0$, and then two objects, u,v∈U, are not related. If $0<R_{A}\left(u,v\right)\leq 1$, then two objects have a valued tolerance relation. If $R_{A}\left(u,v\right)$ is closer to 1, then two objects are more related. Then the valued tolerance relation matrix is built, $\forall \;\left(u,v\right)\in U\times U:\;0\leq R_{A}\left(u,v\right)\leq 1$, where this matrix is symmetric with the main diagonal is equal to 1. This definition supports Definition 4 to calculate the tolerance granule for each object in U.

**Definition** **4.** *A tolerance granule of object* u∈U*for relation* $R_{A}$*is denoted by* $R_{A}\left(u\right)$*and calculated as follows:*

(3) $$R_{A}\left(u\right)=\left\{v\in U\;:R_{A}\left(u,v\right)>0\right\}$$

Definition 4 is used to determine whether the objects in U have the valued tolerance relation with an object, u∈U, built on a set of conditional attributes, A⊆C. Here, $R_{A}\left(u\right)$ is a tolerance granule of *u*, where it carries a set of objects that has the valued tolerance relation with u (i.e., a set of supporting objects of u). This definition supports Definition 5 to determine the lower and upper approximations of the decision classes (i.e., the tolerance classes) built on a set of conditional attributes, A⊆C.

**Definition** **5.** *With a set of decision classes,* D⊆U*, a subset of conditional attributes,* A⊆C*, and an object,* u∈U*, the lower approximations and upper approximations of* D for A *are denoted by* $D_{A}$ *and* $D^{A}$ *and defined as follows:*

(4) $$D_{A}=\left\{\forall u\in U\;:R_{A}\left(u\right)\subseteq D\right\}$$

(5) $$D^{A}=\left\{\forall u\in U\;:R_{A}\left(u\right)\cap D\neq Ø\right\}$$

Definition 5 used is to determine a lower approximation, $D_{A}$, of decision class D, which has tolerance granules, $R_{A}\left(u\right)$, involved in D and an upper approximation, $D^{A}$, of D that has tolerance granules, $R_{A}\left(u\right)$, intersected with D. In this way, any objects that belong to $D_{A}$ are certainly involved in D; meanwhile, any objects that belong to $D^{A}$ can be involved in D or not. Therefore, the determination of the lower and upper approximations plays an important role in the performance of the VTRS–DR method since it strongly affects the decision results, as well as the calculation space. The closer the $D_{A}$ and $D^{A}$ are to the D, the more accurate the results and the lower the computational cost.

**Definition** **6.** *With a setof decision classes,* D⊆U*, a subset of conditional attributes,* A⊆C*, and an object,* u∈U*, the membership degrees of object* u∈U*belonging to* $D_{A}$*and* $D^{A}$*are denoted by* $\mu_{D_{A}}\left(u\right)$*and* $\mu_{D^{A}}\left(u\right)$*and calculated as follows:*

(6) $$\mu_{D_{A}}\left(u\right)=\underset{v\in R_{A}\left(u\right)}{min}\left(max\left(1-R_{A}\left(u,v\right),\;\hat{v}\right)\right)$$

(7) $$\mu_{D^{A}}\left(u\right)=\underset{v\in R_{A}\left(u\right)}{max}\left(min\left(R_{A}\left(u,v\right),\;\hat{v}\right)\right)$$

where $\hat{v}$ is the membership degree of object v in the set D (i.e., $\hat{v}=\{\begin{array}{c} 1,v\in D\\ 0,v\notin D \end{array}$ or $\hat{v}\in \left\{0,\;1\right\}$).

Definition 6 is used to calculate the membership degree of an object, u∈U, to find out whether it belongs to the lower or upper approximation. The $\mu_{D_{A}}\left(u\right)$ and $\mu_{D^{A}}\left(u\right)$ values are in the interval between 0 and 1; thus, the object, u, will belong to the approximation that is closer to 1. This is an important criterion to determine the decision class, D, that the object, u, belongs to.

**Definition** **7.** *With a decision table* DT=(U, C∪{d})*and a subset of attributes,* A⊆C*, a decision rule that describes an object,* $u_{i}\in U$, i=1, 2, …, m*, is denoted by* $\rho_{i}$*, and defined by the following:*

(8) $$\rho_{i}\overset{\mathrm{def}}{=}\underset{RSS_{j}\;\in \;A}{\wedge }\left(RSS_{j}=RSS_{j}\left(u_{i}\right)\right)\to \left(D=d\left(u_{i}\right)\right)$$

where $RSS_{j}\left(u_{i}\right)$ is a value of $RSS_{j}\in A$ and $\left(D=d\left(u_{i}\right)\right)$ is a decision class based on decision attribute d of the object $u_{i}$.

Definition 7 is used to describe a decision rule, $\rho_{i}$, corresponding to an object, $u_{i}\in U$, i=1, 2, …, m. In Equation (8), the left side presents a set of conditional attributes, A⊆C, and the right side presents a decision attribute, d, corresponding to a decision class that involves the object, u.

**Definition** **8.** *With a subset of attributes,* A⊆C*, the credibility degree of a decision rule* $\rho_{i}$, i=1, 2, …, m*, is denoted by* $\mu_{A}\left(\rho_{i}\right)$*and calculated as follows:*

(9) $$\mu_{A}\left(\rho_{i}\right)=\underset{u\in S_{A}\left(\rho_{i}\right)}{min}\left(max\left(1-R_{A}\left(\rho_{i},u\right),\;\mu_{D_{A}}\left(u\right)\right)\right)\;=\underset{u\in R_{A}\left(u_{i}\right)}{min}\left(max\left(1-R_{A}\left(u_{i},\;u\right),\;\mu_{D_{A}}\left(u\right)\right)\right)$$

Definition 8 is to calculate the credibility degree of a decision rule, $\rho_{i}$, i=1, 2, …, m. The $\mu_{A}\left(\rho_{i}\right)$ value is in the interval between 0 and 1. If this value is closer to 1, then it is reliable that the object $u_{i}$ is involved in the decision class D. This is an important criterion for classifying a new object into a decision class.

**Definition** **9.** *With a subset of attributes,* A⊆C*, a support object set of a decision rule* $\rho_{i}$, i=1, 2, …, m*, is denoted by* $S_{A}\left(\rho_{i}\right)$*and calculated as follows:*

(10) $$S_{A}\left(\rho_{i}\right)=\left\{u\in U\;:\;R_{A}\left(\rho_{i},u\right)>0\right\}=\left\{u\in U\;:\;R_{A}\left(u_{i},u\right)>0\right\}=R_{A}\left(u_{i}\right)$$

In Equation (10), $S_{A}\left(\rho_{i}\right)$ is the tolerance granule of rule $\rho_{i}$ for relation $s_{A}$. It means, on binary relation ${\left\{\rho_{i}\right\}}_{i=1}^{m}\times U$, and with a set of conditional attributes, A⊆C, we build a valued tolerance relation, $s_{A}:\;{\left\{\rho_{i}\right\}}_{i=1}^{m}\times U\;\to \left[0,\;1\right]$, such that $s_{A}\left(\rho_{i},u\right)>0$, where $s_{A}\left(\rho_{i},u\right)$ is the support degree of rule $\rho_{i}$ for object u∈U. Object u is similar to some extent to the conditional part of the rule $\rho_{i}$ on the set of conditional attributes, A⊆C. Moreover, $s_{A}$ is a valued tolerance relation defined exactly as the relation $R_{A}$. Thus, $s_{A}\left(\rho_{i},u\right)$ is calculated as in Equation (3), i.e., ∀u∈U and i=1, 2, …, m, $s_{A}\left(\rho_{i},u\right)\equiv R_{A}\left(\rho_{i},u\right)\equiv R_{A}\left(u_{i},u\right)$, where $u_{i}$ is an object of the rule $\rho_{i}$.

Definition 9 is to determine the $S_{A}\left(\rho_{i}\right)$, a support object set of a decision rule, $\rho_{i}$, corresponding to an object, $u_{i}$, i=1, 2, …, m. The $S_{A}\left(\rho_{i}\right)$ of $\rho_{i}$ is equivalent to a tolerance granule, $R_{A}\left(u_{i}\right)$, of the object $u_{i}$. If many decision classes have the same credibility degrees, then it is difficult to decide which class the object $u_{i}$ belongs to; thus, the support object set of each class will help to solve the problem of classification. This value plays an important role in classifying a new object into a decision class.

## 4. The Proposed Positioning Method

**Figure 1:**
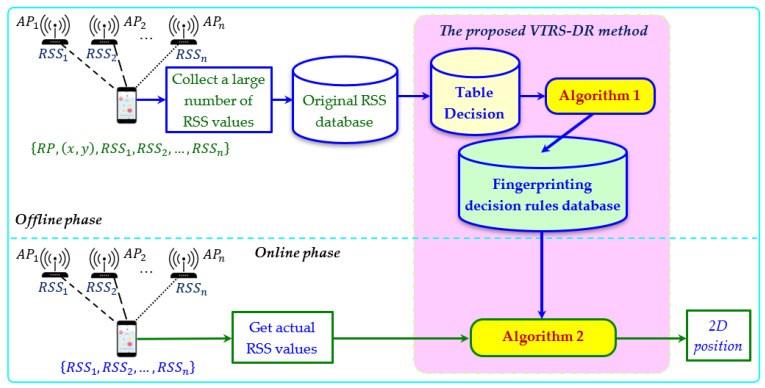
The overall structure of the proposed method.

In this section, to apply the VTRS–DR method to the WiFi fingerprinting method, we recount how we used the VTRS–DR method with a set of conditional attributes, A=C, meaning that the whole set of C (i.e., the whole APs) in the decision table, DT, was applied. Figure 1 shows the overall structure of the proposed method. The method is applied not only in the offline phase but also in the online phase. For the offline phase, at each reference point, $RP_{l}$, l=1, 2, …, N, a large number of RSS values from n APs is collected. Then the original RSS fingerprinting database is created, as shown in Table 2. The structure of this database is presented as follows: {Position, Coordinate, RSS values from APs}. To make this database similar to the decision table, DT, in Table 1, two parts {Position, RSS values from APs} in the original database are copied, and the index of each row is added as the first column to mark the objects. Finally, Algorithm 1 is applied to the DT to make the fingerprinting decision rules database. The structure of the fingerprinting decision rules database is shown in Table 3, with the format as follows: {Rule, Conditions of rule, Decision attribute, Support components}. Here, the support components include two values: the credibility degree and the support object set value. For the online phase, based on the fingerprinting decision rules database and measured RSS values $\theta =\left(\theta_{1},\theta_{2},\ldots ,\theta_{n}\right)$ received from one unknown position, the user’s position is obtained via Algorithm 2. Algorithm 2 chooses the best RP candidate as the user’s position among the whole RPs by considering the valued tolerance relation, the credibility degree, the support object set value, and the Euclidean distance. One example of the proposed method can be seen in Appendix A.
**Algorithm 1:** The Construction of a Fingerprinting Decision Rules Database for IPS**Input:** The decision table DT.
**Output:** The fingerprinting decision rules database.
**Step 1.** Create the valued tolerance relation matrix from relations of all pairs of two objects (two rows) in DT: $R_{A}\left(u,v\right)$, ∀u,v∈U (Equation (1) and Equation (2)).
**Step 2.** Determine the tolerance granule, $R_{A}\left(u\right)$, ∀u∈U (Equation (3)).
**Step 3.** Determine the set of decision classes D (i.e., a total number of RPs) of decision table, DT, based on the decision attribute, d. For each decision class, $D_{l}$, calculate the lower approximations, ${\left(D_{l}\right)}_{A}$ and the upper approximations, ${\left(D_{l}\right)}^{A}$, l=1, 2, …, N (Equation (4) and Equation (5)).
**Step 4.** Calculate the membership degrees, $\mu_{{\left(D_{l}\right)}_{A}}\left(u\right)$ and $\mu_{{\left(D_{l}\right)}^{A}}\left(u\right)$, of object u, corresponding to ${\left(D_{l}\right)}_{A}$ and ${\left(D_{l}\right)}^{A}$, $\forall u\in U,\;\;\forall D_{l}\in \left\{D_{1},\;D_{2},\;\ldots ,D_{N}\right\}\equiv \left\{RP_{1},\;RP_{2},\;\ldots ,RP_{N}\right\}$ (Equation (6) and Equation (7)).
**Step 5.** Define the decision rule, $\rho_{i}$, that describes the object, $u_{i}\in U$, on the set of attributes A, i=1, 2, …, m (Equation (8)).
**Step 6.** Calculate the credibility degree, $\mu_{A}\left(\rho_{i}\right)$, of a decision rule, $\rho_{i}$, i=1, 2, …, m (Equation (9)).
**Step 7.** Define the support object set, $S_{A}\left(\rho_{i}\right)$, of a decision rule, $\rho_{i}$, for the set of attributes A, i=1, 2, …, m (Equation (10)).
**Step 8.** Create the fingerprinting decision rules database.

**Algorithm 2:** The Advanced Positioning Algorithm Based on the Proposed VTRS–DR Method**Input:** Set of measured RSS values, θ, fingerprinting decision rules database.
**Output:** Position of the user.
**Step 1.** Calculate the valued tolerance relation between θ to conditions of rule $\rho_{i}$: $R_{A}\left(\theta ,\rho_{i}\right)$, i=1, 2, …, m (i.e., $R_{A}\left(\theta ,\rho_{i}\right)=\underset{RSS_{j}\;\in \;A}{\min}\left(R_{j}\left(\theta ,\rho_{i}\right)\right)$, as in Equation (2)).
**Step 2.** Determine the smallest value $\mu_{\rho_{i}}\left(\theta \right)$ between the valued tolerance relation $R_{A}\left(\theta ,\rho_{i}\right)$ and the credibility degree of rule $\rho_{i}$: $\mu_{\rho_{i}}\left(\theta \right)=min\left(R_{A}\left(\theta ,\rho_{i}\right),\mu_{A}\left(\rho_{i}\right)\right)$, i=1, 2, …, m.
**Step 3.** Calculate the credibility degree of θ to each decision class $D_{l}$ (i.e., one RP), l=1, 2, …, N: $\mu_{D_{l}}\left(\theta \right)=\underset{\rho_{i}\in R\left(D_{l}\right)}{\max}\left(\mu_{\rho_{i}}\left(\theta \right)\right)$, where $R\left(D_{l}\right)$ is the set of all decision rules that belongs to $D_{l}$ and having $\mu_{\rho_{i}}\left(\theta \right)>0$, i=1, 2, …, m.
**Step 4.** Choose the class $D_{l}$ that maximizes the credibility degree $\mu_{D_{l}}\left(\theta \right)$. If $\exists !D_{l}\;:\;\mu_{D_{l}}\left(\theta \right)$ is at the maximum, then choose this $D_{l}$ as the user’s position.
**Step 5.** If the same maximum credibility degrees exist on different L (L<N) decision classes, $\left\{D_{l_{1}},D_{l_{2}},\ldots ,D_{l_{L}}\right\}$, then calculate the relative supports of rules in $\left\{D_{l_{1}},D_{l_{2}},\ldots ,D_{l_{L}}\right\}$, as denoted by $Supp\left(\rho_{i}\right)$. Note that $Supp\left(\rho_{i}\right)$ is a ratio of the number of the support object set $S_{A}\left(\rho_{i}\right)$ to the total number of the set of all objects in any decision class which has the rule $\rho_{i}$.
**Step 6.** Calculate the aggregated support of θ as $Supp_{D_{l_{p}}}\left(\theta \right)=\underset{\rho_{i}\in R\left(D_{l_{p}}\right)}{\max}\left(Supp\left(\rho_{i}\right)\right)$, p=1, 2, …, L.
**Step 7.** Choose the $D_{l_{p}}$ that maximizes the aggregated support $Supp_{D_{l_{p}}}\left(\theta \right)$. If $\exists !D_{l_{p}}\;:\;Supp_{D_{l_{p}}}\left(\theta \right)$ is at the maximum, then choose this $D_{l_{p}}$ as the user’s position.
**Step 8.** If the same maximum aggregated support values, $Supp_{D_{l_{p}}}\left(\theta \right)$, exist on many decision classes, then use the Euclidean distance to choose the decision class $D_{l_{p}}$ which has the minimum distance between the set of measured RSS values θ and the mean of RSS values of that decision class; then choose that class (i.e., one RP) as the user’s position.

## 5. Experimental Results

### 5.1. Experimental Setup

**Figure 2:**
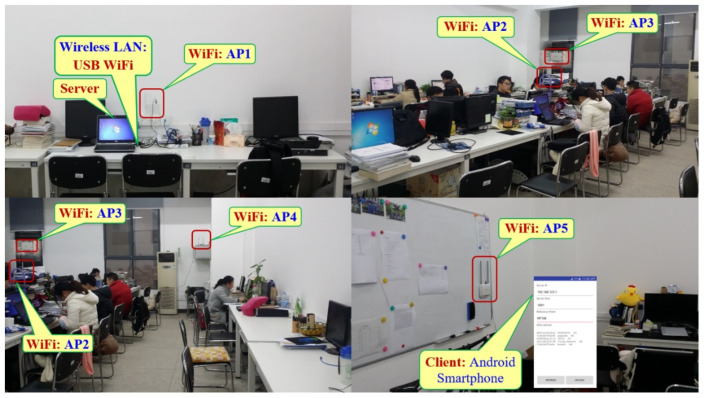
The office room for data collection.

**Figure 3:**
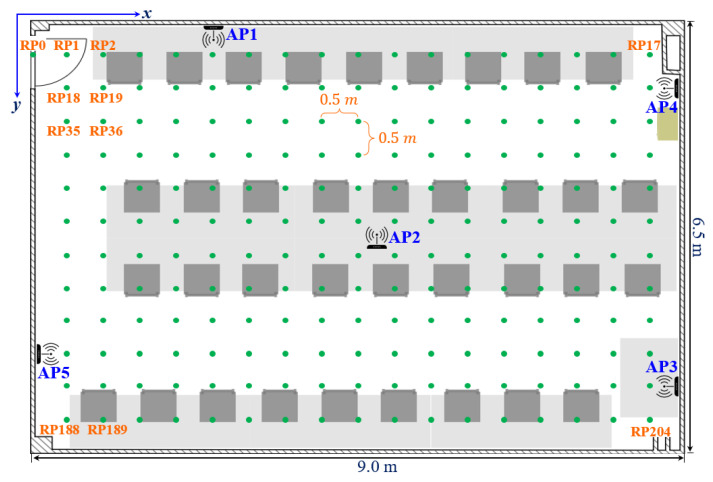
The positions of five APs and 205 APs in the office room.

To validate the efficiency and accuracy of the proposed method, we used the dataset introduced by Duong-Bao et al. [39]. The dataset was created in an office room at Hunan University, China, over four months. The room had an area of 9.0 × 6.5 m^2^. Five APs were installed at different positions, and each one was fixed at 1.1 m to 1.6 m from the ground, as can be seen in Figure 2. The smartphone played a role as a client to send the scanned RSS values to the server (i.e., the laptop). This server was responsible for storing the RSS values, creating the radio map, and classifying the user’s position among predefined RPs. Figure 3 shows the positions of the APs, as well as the RPs in a two-dimensional coordinate. From this figure, there existed 205 RPs in the room, and the grid spacing between two adjacent RPs was 0.5 m. The starting point (RP0) was marked at the entrance door on the top left of the figure, and the other RPs were marked orderly from the left side to the right side, and from the top to the bottom. The last point (RP204) ended at the bottom right, where it was nearby the AP3.

In the offline phase, a subject holding a smartphone in front of his body stood at each determined RP to collect the RSS values from the five available APs. For each RP, the subject scanned the RSS values 100 times in different directions, meaning that there were 20,500 scanning times for 205 RPs. In this phase, various environmental conditions (i.e., the density of people and electrical devices, the direction of the user, the period of a day, etc.) were considered to create a noisy and complex fingerprinting database. In the online phase, the RSS values were collected in two separate test cases which were set up with different environmental conditions. Table 4 shows the conditions for the RSS collection of the two cases. For each case, the subject stood on each RP and used the smartphone to scan the RSS values once, meaning that the subject collected the RSS values 205 times over 205 RPs (i.e., 410 times in two cases). More detailed descriptions of the dataset can be found in Reference [31].

### 5.2. Experimental Results

From the aforementioned dataset, in this experiment, the fingerprinting decision rules database was created from the original RSS fingerprinting database, which involves the RSS values collected from 205 RPs in the offline phase. Therefore, the training data used to build the new database include 20,500 objects corresponding to 20,500 samples (i.e., 20,500 rows) and five conditional attributes corresponding to five APs (i.e., five columns). At each RP position, the subject collected the RSS values 100 times; thus, there are 100 rules for each class (i.e., each RP), which also means that, for each RP, there are 100 collected fingerprints (i.e., set of RSS values). The testing data used the two test cases that were collected in the online phase of the above dataset to classify the user’s position. For each test case, the RSS values from the five APs were collected at each RP once, meaning there were 205 sets of RSS values collected over 205 RPs used for performance evaluation.

The discrimination thresholds, k, corresponding to the number of conditional attributes are shown in Table 5. To the best of our knowledge, the *k* values are mostly chosen from the trial-and-error method, meaning that different sets of k values were put into the proposed method and repeatedly tested to find out the best k values. We chose the discrimination threshold values, $k_{j}$, for each attribute $RSS_{j}$, j=1, 2, …, n, to measure the similarity among the objects and calculate the experimental results to find out which set of $k_{j}$ can help the VTRS–DR method to achieve the best result. With each set of $k_{j}$, the VTRS–DR method builds a fingerprinting decision rules database. We used the database with 20,500 rows to test with the fingerprinting decision rules database corresponding to each k. From the classification results corresponding to different sets of k values, we then chose the set that maximizes the result.

To evaluate the performance of our proposed method, we compared it with the NN-based methods (i.e., the 1-NN and WKNN (K is chosen as 3)) [17] and the random statistical (RS) method [14]. Note that the NN-based methods use the original fingerprinting database and the RS method uses the standardized fingerprinting database; meanwhile, the proposed method uses the fingerprinting decision rule database when matching with the measured RSS values in the online phase. For the compared methods, in the offline phase, the fingerprint of one RP is presented as a set of means of collected RSS values (i.e., 100 RSS values for each available AP at that RP). In the online phase, the NN-based and RS methods calculate the distance between the measured RSS values with the fingerprints of the whole RPs to find out the user’s position. The Euclidean distance was used for the two NN-based methods, while the Mahalanobis distance was used for the RS method, respectively. The mean error in the experiment is calculated as follows:

(11) $$\Delta_{ME}=\frac{\sum_{l=1}^{N}E_{l}}{N}$$

where $\Delta_{ME}$ is the mean error (m), N is the number of RPs used in the online phase (N=205), and $E_{l}$ is the Euclidean distance between $RP_{l}$ and its classified position.

**Figure 4:**
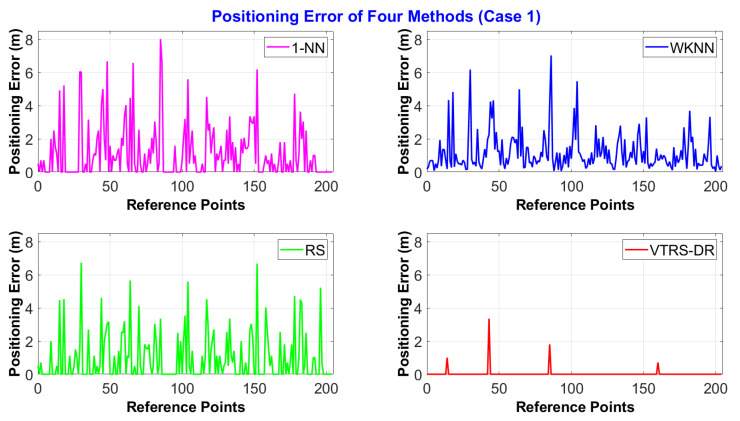
The positioning error distance of four methods via 205 RPs in Case 1.

Figure 4 shows the results of positioning errors of the four methods for the dataset of Case 1. It can be seen from the figure that the VTRS–DR method achieves the best results, as it can mostly find out the exact user’s positions among the predefined RPs (i.e., the same positions as the RPs where the user stood), which means no error (0 m). In Figure 4, there are only four positions (the 15th, 44th, 86th, and 162nd positions) where the proposed method obtains the wrong results, while the 1-NN and RS methods obtain 93 and 113 wrong results. The positioning errors corresponding to the four wrong estimated positions are 1 m, 3.35 m, 1.80 m, and 0.71 m, respectively. Comparing the mean positioning errors, the 1-NN, WKNN, and RS methods achieve errors around 1 m, which are 1.13 m for 1-NN, 1.17 m for WKNN, and 0.94 m for RS, respectively. Meanwhile, the mean errors of the proposed method are far better than others with only 0.033 m. A deeper statistical comparison of error positions is pointed out in Table 6. In this table, we can see that the WKNN method has the smallest minimum positioning error, with only 0.05 m, while the maximum error of the VTRS–DR method is only 3.35 m, which is approximately half of that of other methods, with 8.02, 7.03 m, and 6.73 m, respectively. However, the mean positioning error at wrong classified positions of the proposed method is not the smallest one (1.71 m with the standard deviation of 1.03 m), as it is higher than the WKNN mean error of only 1.17 m but lower than the two others, 2.05 m and 2.06 m. The results show the superiority of the VTRS–DR method to other methods for the dataset of Case 1 since it improves the classified accuracy from the above methods by 53.17% and 43.42%. The WKNN method cannot provide the exact positions where the user stood since this method calculates the mean positions from the three nearest candidates.

**Figure 5:**
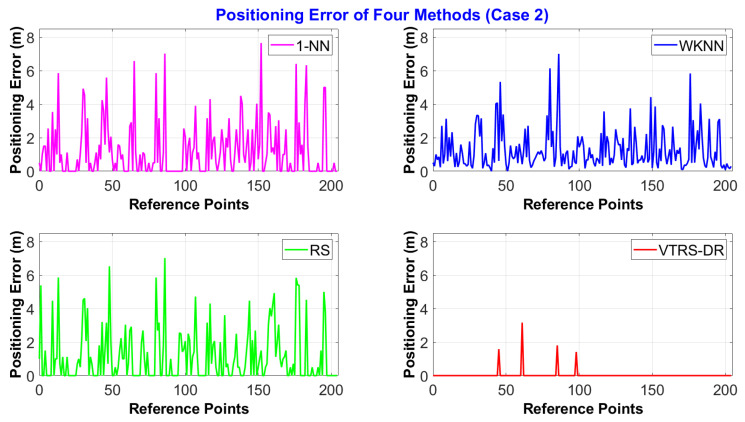
The positioning error distance of four methods via 205 RPs in Case 2.

Figure 5 displays the positioning errors of the four methods for the dataset of Case 2, where the environmental conditions were set up as being more complicated, such as increasing the number of working people or increasing the number of electrical devices. Overall, even though the testing condition is more complex, the proposed method is still better than others since it still maintains a very small number of wrong estimated positions with only four errors (the 46th, 62nd, 86th, and 99th positions) compared to the 1-NN (116) and RS (108) methods, meaning that it reduces the classification errors by 54.64% and 50.73%, respectively. The positioning errors corresponding to the four wrong estimated positions are 1.58 m, 3.16 m, 1.80 m, and 1.41 m, respectively. The mean positioning error of the proposed method is the best one with only 0.039 m, while the others’ errors are around 1.2 m. Table 7 shows the further statistical analysis of the four methods in Case 2 at error positions. From this table, the proposed method still achieves the smallest maximum error with 3.16 m, but the mean positioning error of the WKNN method is higher than others with 1.26 m.

**Figure 6:**
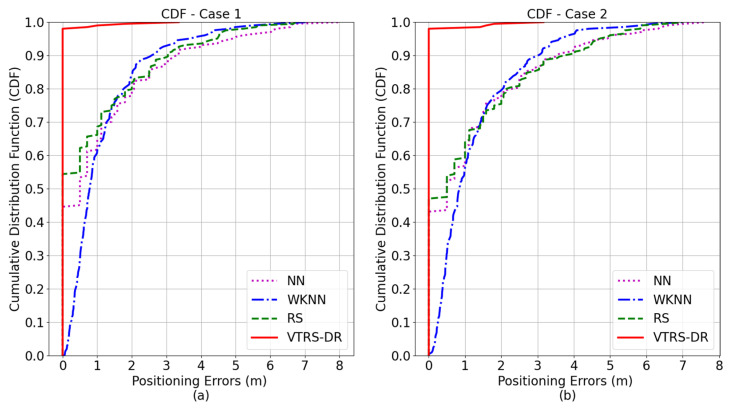
Cumulative position error distributions of the four methods in (**a**) Case 1 and (**b**) Case 2.

Moreover, the cumulative position error distributions in the two test cases are shown in Figure 6. For Case 1 (a), at the 60th percentile, the positioning error of the proposed method is 0 m, and at the 90th percentile, the error is still 0 m. The 0 m error continues until the 98th percentile. At the same percentiles, the errors of the NN, the WKNN, and RS methods are 0.68 m, 0.48 m, and 0.93 m at the 60th percentile and 3.24 m, 3.02 m, and 2.61 m at the 90th percentile, respectively. From this result, the VTRS–DR method certainly achieves much better accuracy compared to others. For Case 2 (b), the same phenomenon for the positioning errors among four methods also happens where the error of the proposed method is greatly smaller than the results of the other methods at the same percentiles. From both test cases, the VTRS–DR method outperforms the performance of the other methods from the point of view of classification since it can help to correctly classify the unknown position among the predefined RPs. The major reason for the extraordinary results of the proposed method is that it uses many classification levels (i.e., calculating the valued tolerance relation, the credibility degree, the support object set value, and the Euclidean distance).

**Figure 7:**
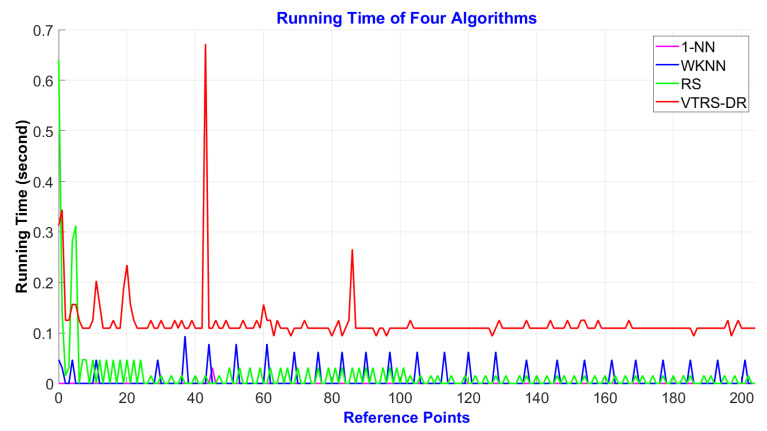
The running time of four methods via 205 RPs in Case 1.

The computational cost of the four implemented methods is analyzed by calculating the running time of each method on MATLAB (R2019a). We used a Dell Vostro laptop which was equipped with an Intel(R) Core (TM) i7-8550U CPU @1.80 GHz processor, 8 GB RAM, and 128 GB SSD. The operating system was Microsoft Windows 10 Professional Edition (64-bit). The computational cost for creating the fingerprinting decision rules database in the offline phase is approximately 96.62 min, and the time for creating the valued tolerance relation matrix obtains 89.34% of the total running time. Furthermore, Figure 7 shows the running time of the four algorithms over 205 RPs in Case 1 to classify the user’s position in the online phase. The figure revealed that the 1-NN becomes the smallest average cost of about 0.0002 s while the VTRS–DR method obtains the biggest average cost of about 0.12 s. Concurrently, the WKNN and RS methods obtain the average values of 0.0077 and 0.0155 s, respectively. From the above costs in two phases, we can easily see that the proposed method will take lots of time to build the fingerprinting decision rules databases, especially the valued tolerance relation matrix, but it does not take much time to estimate the user’s position in the online phase. This means that the proposed method can be used in the real-time scenario, while the database creation can be performed on the server readily.

Furthermore, to evaluate the robustness of the proposed method, we intentionally reduced the number of APs to four and three APs instead of using the default five APs. Since there are many possible sets of using four or three APs, we used the combination method to consider the whole possible cases. Therefore, if we consider three APs, the number of possible cases is $C_{5}^{3}=10$, and with four APs, this number is $C_{5}^{4}=5$, respectively. The positioning results of the proposed method in two test cases were also compared to the 1-NN, WKNN, and RS methods. Table 8 and Table 9 show the mean positioning errors among the four methods in two cases with different combinations of four APs. For example, the notation {1, 2, 3, 4} in the tables represents the combination of four APs from 1 to 4. From both tables, even though the mean positioning errors of all cases increase, the VTRS–DR method still shows the best performance. The error of the proposed method is by far much lower than the others.

**Figure 8:**
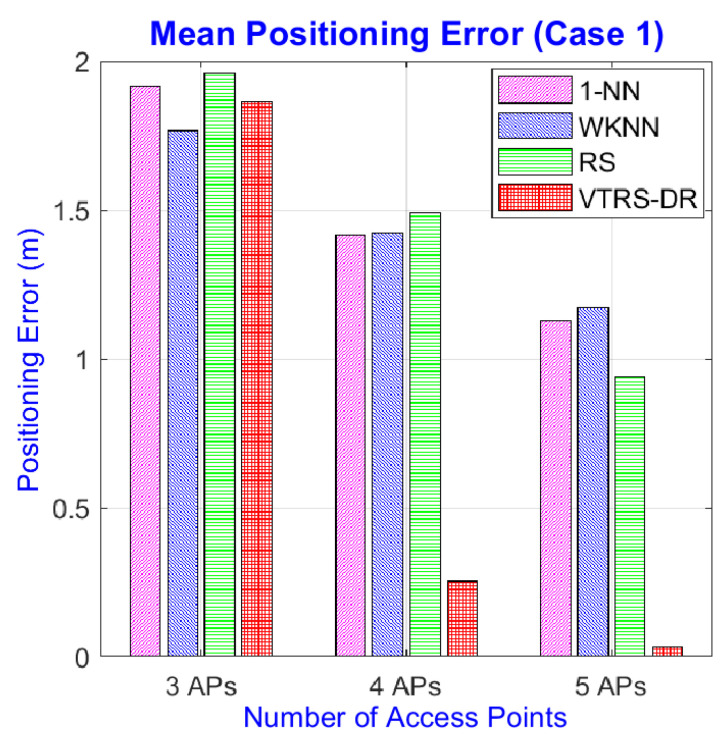
Mean of mean errors of four methods with different numbers of APs in Case 1.

**Figure 9:**
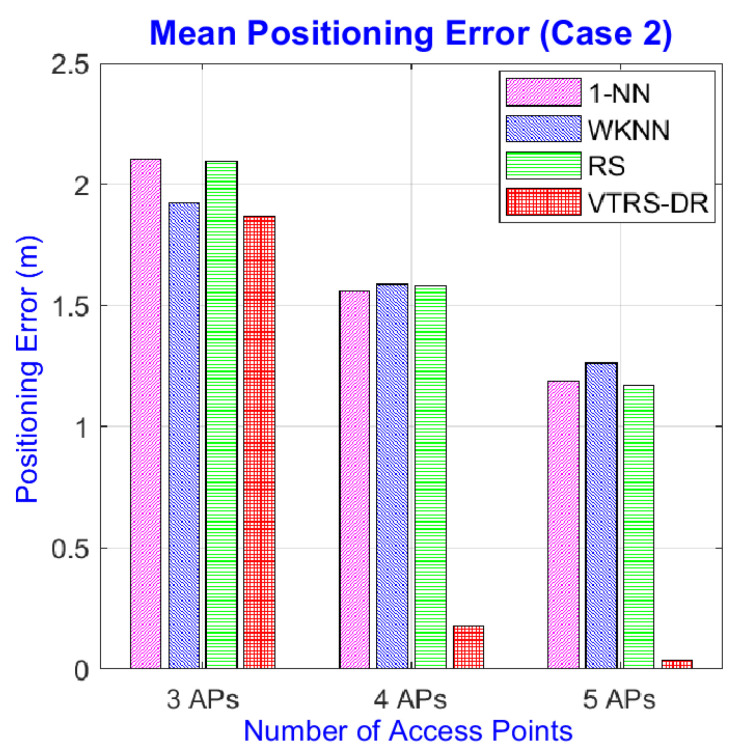
Mean of mean errors of four methods with different numbers of APs in Case 2.

However, this is not right when using only three APs in two test cases. The mean positioning errors can be seen in Table 10 and Table 11. The results show that the VTRS–DR method cannot outperform the positioning results of other methods; even some combinations’ results of the proposed method are much worse than others, for example, the cases of {1, 3, 4} in both cases. The reason can come from the characteristic of the VTRS–DR method that it may require more than a certain number of attributes (i.e., the number of APs) to produce good results. This problem, however, might not occur in practical scenes due to the widespread of APs indoors, especially in urban areas. Figure 8 and Figure 9 display the comparisons of the mean of mean errors of the four methods in three different numbers of APs. From these figures, we can see that the mean of mean errors of the VTRS–DR methods is much better than others in cases using five and four APs, but for three APs, it is slightly better than others in Case 2 but somewhat worse than WKNN method in Case 1 (i.e., 1.766 m for WKNN and 1.865 m for VTRS–DR). Meanwhile, the RS method obtains the worst error in Case 1 with 1.959 m, and the 1-NN method gets the worst error in Case 2 with 2.103 m. The accuracy of the proposed method in Case 2 is better than in Case 1; we think that this is because the RSS values in Case 1 are more stable at any RPs than in Case 2 due to their simpler setup; thus, there may exist many valued tolerance relations that have the same values, and this makes it hard to classify the decision classes clearly. Therefore, the proposed method can work well in complicated environments, which are more popular in real life than stable environments are, such as Case 1.

## 6. Conclusions

In this paper, a novel WiFi fingerprinting positioning method based on the valued tolerance and decision rules method was proposed. The proposed method is implemented not only in the offline phase but also in the online phase. In the offline phase, the fingerprinting decision rules database is built from the decision table based on the conventional RSS fingerprinting database. The created database consists of different components, such as the credibility degree and support object set, which are utilized to support the classification of the measured RSS values in the online phase to the precise class. This helps to increase the classification performance. The evaluation shows that the proposed method outperforms the other methods for both the datasets of the two cases which were set up with different environmental conditions, although the test data were collected in a small number (only two sets) of RSS samples on the exact position of each RP rather than the random positions in the target area. The wrong classification rate of the proposed method is very low, which reduced the errors from the other methods from 43.42% to 54.64%. However, the proposed method also has some limitations. The first one is high complexity. Even though it can achieve extraordinary positioning results, the computational complexity makes it difficult to be used in real-time since it needs to compute the relation matrix in the offline phase, as well as the complicated components, such as the credibility degree and support object set values in the online phase. The second limitation is that the performance of the proposed method will greatly degrade when the number of available APs is reduced. For future work, we want to evaluate the performance of this method in bigger areas, such as buildings with multiple floors and various available APs that consist of more complicated environmental conditions. Furthermore, we also want to investigate how to optimize the number, as well as the setup positions, of APs in a target area.

## Figures and Tables

**Table 1:** The structure of the decision table.

U	$RSS_{1}\;(\mathrm{dBm})$	$RSS_{2}\;(\mathrm{dBm})$	…	$RSS_{n}\;(\mathrm{dBm})$	d
$u_{1}$	−51	−54	…	−52	$RP_{1}$
$u_{2}$	−46	−47	…	−51	$RP_{2}$
$u_{3}$	−50	−56	…	−53	$RP_{1}$
⋮	⋮	⋮	⋮	⋮	⋮
$u_{m-1}$	−76	−43	…	−41	$RP_{N}$
$u_{m}$	−73	−41	…	−38	$RP_{N}$

**Table 2:**
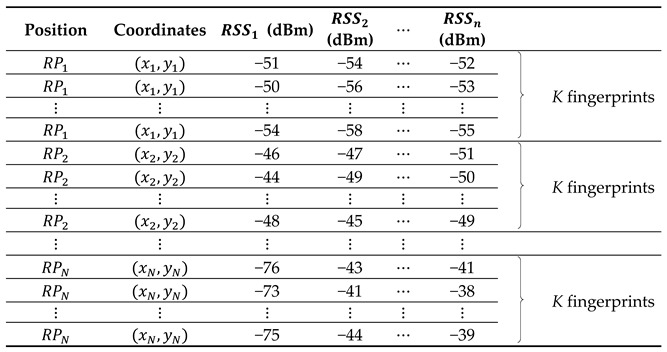
The original RSS database.

**Table 3:**
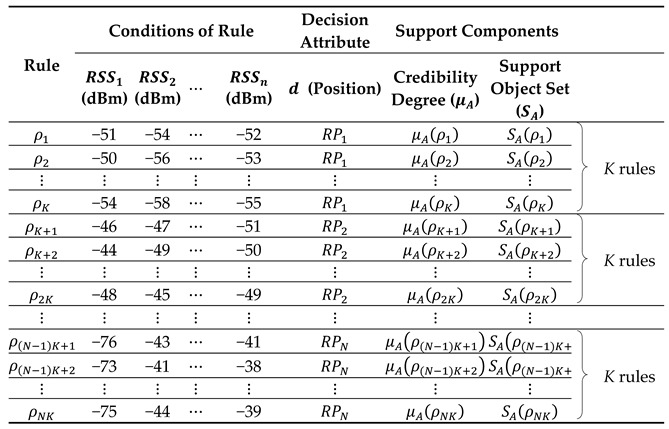
The structure of the fingerprinting decision rules database.

**Table 4:** The difference in environmental conditions in Case 1 and Case 2.

Conditions	Case 1	Case 2
Density of people	1 to 9	6 to 13
Density of electrical devices	11	20
Temperature	Cool	Warm
Height from the ground	1.3 m	1.3 m
Subject direction	Random	Random

**Table 5:** The discrimination threshold values, k.

Attribute	$RSS_{1}$	$RSS_{2}$	$RSS_{3}$	$RSS_{4}$	$RSS_{5}$
$k_{j}$	1.97	1.98	1.96	1.95	1.99

**Table 6:** Statistical comparison of four methods in Case 1 at error positions.

	1-NN	WKNN	RS	VTRS–DR
No. of Errors	113	205	93	**4**
Max (m)	8.02	7.03	6.73	**3.35**
Min (m)	0.50	**0.05**	0.50	0.71
Mean (m)	2.05	**1.17**	2.06	1.71
Stdev (m)	1.69	1.15	1.52	**1.03**

**Table 7:** Statistical comparison of four methods in Case 2 at error positions.

	1-NN	WKNN	RS	VTRS–DR
No. of Errors	116	205	108	**4**
Max (m)	7.65	7.00	7.02	**3.16**
Min (m)	0.50	**0.001**	0.50	1.41
Mean (m)	2.10	**1.26**	2.21	1.99
Stdev (m)	1.67	1.20	1.62	**0.69**

**Table 8:** Mean errors of using four APs in Case 1.

Case 1 (4 APs)	{1, 2, 3, 4}	{1, 2, 3, 5}	{1, 2, 4, 5}	{1, 3, 4, 5}	{2, 3, 4, 5}	Mean (m)
1-NN	1.374	1.433	1.411	1.471	1.390	1.416
WKNN	1.352	1.346	1.532	1.371	1.514	1.423
RS	1.588	1.350	1.407	1.462	1.636	1.489
VTRS–DR	**0.210**	**0.184**	**0.315**	**0.227**	**0.334**	**0.254**

**Table 9:** Mean errors of using four APs in Case 2.

Case 2 (4 APs)	{1, 2, 3, 4}	{1, 2, 3, 5}	{1, 2, 4, 5}	{1, 3, 4, 5}	{2, 3, 4, 5}	Mean (m)
1-NN	1.527	1.536	1.732	1.473	1.520	1.558
WKNN	1.599	1.537	1.735	1.496	1.563	1.586
RS	1.588	1.538	1.663	1.477	1.650	1.583
VTRS–DR	**0.183**	**0.116**	**0.237**	**0.191**	**0.174**	**0.180**

**Table 10:** Mean errors of using three APs in Case 1.

Case 1 (3 APs)	{1, 2, 3}	{1, 2, 4}	{1, 2, 5}	{1, 3, 4}	{1, 3, 5}	{1, 4, 5}	{2, 3, 4}	{2, 3, 5}	{2, 4, 5}	{3, 4, 5}	Mean (m)
1-NN	1.845	1.858	2.000	1.999	1.713	1.902	2.011	1.716	2.230	1.878	1.915
WKNN	1.679	**1.843**	1.778	**1.741**	1.532	**1.721**	**1.864**	1.715	2.050	1.732	**1.766**
RS	1.785	1.930	1.879	1.996	1.643	1.821	2.138	1.977	2.338	2.089	1.959
VTRS–DR	**1.478**	1.848	**1.650**	3.486	**1.237**	1.810	2.004	**1.444**	**2.024**	**1.664**	1.865

**Table 11:** Mean errors of using three APs in Case 2.

Case 2 (3 APs)	{1, 2, 3}	{1, 2, 4}	{1, 2, 5}	{1, 3, 4}	{1, 3, 5}	{1, 4, 5}	{2, 3, 4}	{2, 3, 5}	{2, 4, 5}	{3, 4, 5}	Mean (m)
1-NN	1.912	2.120	2.294	2.133	1.993	2.125	2.159	2.001	2.418	1.873	2.103
WKNN	1.793	1.975	2.028	**1.798**	1.740	2.018	1.934	1.929	2.234	1.749	1.920
RS	1.821	2.112	2.292	1.954	1.925	2.137	2.165	2.058	2.521	1.947	2.093
VTRS–DR	**1.447**	**1.828**	**1.735**	3.390	**1.461**	**1.733**	**1.921**	**1.631**	**1.920**	**1.597**	**1.866**

## Data Availability

The data for this article can be found in the online version at https://github.com/luongnt1983/IPS (accessed on 15 June 2022).

## Appendix A

In this part, we provide an example of how to use the VTRS–DR method to determine the user’s position. Suppose we set up five APs and there are ten predefined *RP*s, $RP_{1},RP_{2},\ldots ,RP_{10}$, in a test area. At each RP, we collected the RSS values twice, as in Table A1. The decision table, DT, was created from Table A1, as shown in Table A2.

**Table A1:** The original RSS database proposed for the example.

Position	Coordinates	$RSS_{1}\;(\mathrm{dBm})$	$RSS_{2}\;(\mathrm{dBm})$	$RSS_{3}\;(\mathrm{dBm})$	$RSS_{4}\;(\mathrm{dBm})$	$RSS_{5}\;(\mathrm{dBm})$
$RP_{1}$	$\left(x_{1},y_{1}\right)$	−57	−48	−64	−45	−52
$RP_{1}$	$\left(x_{1},y_{1}\right)$	−58	−49	−63	−48	−53
$RP_{2}$	$\left(x_{2},y_{2}\right)$	−57	−43	−65	−51	−53
$RP_{2}$	$\left(x_{2},y_{2}\right)$	−58	−42	−67	−51	−55
$RP_{3}$	$\left(x_{3},y_{3}\right)$	−55	−54	−63	−52	−57
$RP_{3}$	$\left(x_{3},y_{3}\right)$	−56	−51	−62	−52	−58
$RP_{4}$	$\left(x_{4},y_{4}\right)$	−40	−50	−57	−52	−53
$RP_{4}$	$\left(x_{4},y_{4}\right)$	−43	−51	−61	−53	−53
$RP_{5}$	$\left(x_{5},y_{5}\right)$	−41	−53	−62	−39	−50
$RP_{5}$	$\left(x_{5},y_{5}\right)$	−42	−53	−63	−39	−49
$RP_{6}$	$\left(x_{6},y_{6}\right)$	−50	−49	−61	−53	−50
$RP_{6}$	$\left(x_{6},y_{6}\right)$	−50	−50	−61	−53	−51
$RP_{7}$	$\left(x_{7},y_{7}\right)$	−50	−56	−65	−45	−48
$RP_{7}$	$\left(x_{7},y_{7}\right)$	−51	−56	−69	−46	−49
$RP_{8}$	$\left(x_{8},y_{8}\right)$	−57	−46	−65	−48	−56
$RP_{8}$	$\left(x_{8},y_{8}\right)$	−58	−47	−64	−50	−55
$RP_{9}$	$\left(x_{9},y_{9}\right)$	−59	−49	−63	−47	−56
$RP_{9}$	$\left(x_{9},y_{9}\right)$	−60	−51	−64	−47	−54
$RP_{10}$	$\left(x_{10},y_{10}\right)$	−67	−51	−66	−51	−42
$RP_{10}$	$\left(x_{10},y_{10}\right)$	−66	−53	−66	−51	−43

**Table A2:** The structure of the decision table created from the original RSS database.

U	$RSS_{1}\;(\mathrm{dBm})$	$RSS_{2}\;(\mathrm{dBm})$	$RSS_{3}\;(\mathrm{dBm})$	$RSS_{4}\;(\mathrm{dBm})$	$RSS_{5}\;(\mathrm{dBm})$	d (Position)
$u_{1}$	−57	−48	−64	−45	−52	$RP_{1}$
$u_{2}$	−58	−49	−63	−48	−53	$RP_{1}$
$u_{3}$	−57	−43	−65	−51	−53	$RP_{2}$
$u_{4}$	−58	−42	−67	−51	−55	$RP_{2}$
⋮	⋮	⋮	⋮	⋮	⋮	⋮
$u_{19}$	−67	−51	−66	−51	−42	$RP_{10}$
$u_{20}$	−66	−53	−66	−51	−43	$RP_{10}$

From Definition 1, the objects that have the valued tolerance relation can belong to the same tolerance class (i.e., decision class). Thus, we calculated the valued tolerance relation for all pairs of objects in the decision table, DT (Definition 3). To keep the generality, we choose A=C and u,v∈U. The valued tolerance relation was calculated from Equations (1) and (2). In this example, we chose $k_{j}$ corresponding to each attribute $RSS_{j}$, j=1, 2, …, 5, based on the standard normal distribution. Then we applied Definition 3 to calculate the valued tolerance relation for all pairs of objects in the decision table, DT (Table 2). For example, we calculated the relation between the two objects $u_{3}$ and $u_{4}$.

$$R_{A}\left(u_{3},u_{4}\right)=min\left\{R_{1}\left(u_{3},u_{4}\right),R_{2}\left(u_{3},u_{4}\right),R_{3}\left(u_{3},u_{4}\right),R_{4}\left(u_{3},u_{4}\right),R_{5}\left(u_{3},u_{4}\right)\right\}\;=min\left\{0.868,\;0.725,\;0.213,\;1.0,\;0.517\right\}=0.213$$

By applying the same way to all pairs, we calculated the valued tolerance relation for 20 objects in Table A2 and finally obtained the valued tolerance relation matrix, as shown in Table A3.

**Table A3:** Valued tolerance relation matrix, $R_{A}$.

$R_{A}$	$u_{1}$	$u_{2}$	$u_{3}$	$u_{4}$	$u_{5}$	$u_{6}$	$u_{7}$	$u_{8}$	$u_{9}$	$u_{10}$	$u_{11}$	$u_{12}$	$u_{13}$	$u_{14}$	$u_{15}$	$u_{16}$	$u_{17}$	$u_{18}$	$u_{19}$	$u_{20}$
$u_{1}$	1.0	0.274	0	0	0	0	0	0	0	0	0	0	0	0	0.034	0	0.034	0.174	0	0
$u_{2}$	0.274	1.0	0	0	0	0	0	0	0	0	0	0	0	0	0.174	0.449	0.275	0.449	0	0
$u_{3}$	0	0	1.0	**0.213**	0	0	0	0	0	0	0	0	0	0	0.174	0	0	0	0	0
$u_{4}$	0	0	**0.213**	1.0	0	0	0	0	0	0	0	0	0	0	0	0	0	0	0	0
$u_{5}$	0	0	0	0	1.0	0.174	0	0	0	0	0	0	0	0	0	0	0	0	0	0
$u_{6}$	0	0	0	0	0.174	1.0	0	0	0	0	0	0	0	0	0	0	0	0	0	0
$u_{7}$	0	0	0	0	0	0	1.0	0	0	0	0	0	0	0	0	0	0	0	0	0
$u_{8}$	0	0	0	0	0	0	0	1.0	0	0	0.073	0.073	0	0	0	0	0	0	0	0
$u_{9}$	0	0	0	0	0	0	0	0	1.0	0.606	0	0	0	0	0	0	0	0	0	0
$u_{10}$	0	0	0	0	0	0	0	0	0.606	1.0	0	0	0	0	0	0	0	0	0	0
$u_{11}$	0	0	0	0	0	0	0	0.073	0	0	1.0	0.725	0	0	0	0	0	0	0	0
$u_{12}$	0	0	0	0	0	0	0	0.073	0	0	0.725	1.0	0	0	0	0	0	0	0	0
$u_{13}$	0	0	0	0	0	0	0	0	0	0	0	0	1.0	0	0	0	0	0	0	0
$u_{14}$	0	0	0	0	0	0	0	0	0	0	0	0	0	1.0	0	0	0	0	0	0
$u_{15}$	0.034	0.174	0.174	0	0	0	0	0	0	0	0	0	0	0	1.0	0.516	0.174	0	0	0
$u_{16}$	0	0.449	0	0	0	0	0	0	0	0	0	0	0	0	0.516	1.0	0.274	0	0	0
$u_{17}$	0.034	0.275	0	0	0	0	0	0	0	0	0	0	0	0	0.174	0.274	1.0	0.449	0	0
$u_{18}$	0.174	0.449	0	0	0	0	0	0	0	0	0	0	0	0	0	0	0.449	1.0	0	0
$u_{19}$	0	0	0	0	0	0	0	0	0	0	0	0	0	0	0	0	0	0	1.0	0.449
$u_{20}$	0	0	0	0	0	0	0	0	0	0	0	0	0	0	0	0	0	0	0.449	1.0

Twenty tolerance granules of object u∈U for relation $R_{A}$ were calculated based on Definition 4, as follows:

$$\begin{array}{ll} R_{A}\left(u_{1}\right)=\left\{u_{1},u_{2},u_{15},u_{17},u_{18}\right\};\; & R_{A}\left(u_{2}\right)=\left\{u_{1},u_{2},u_{15},u_{16},u_{17},u_{18}\right\};\;\\ R_{A}\left(u_{3}\right)=\left\{u_{3},u_{4},u_{15}\right\};\; & R_{A}\left(u_{4}\right)=\left\{u_{3},u_{4}\right\};\\ R_{A}\left(u_{5}\right)=\left\{u_{5},u_{6}\right\};\; & R_{A}\left(u_{6}\right)=\left\{u_{5},u_{6}\right\};\;\\ R_{A}\left(u_{7}\right)=\left\{u_{7}\right\};\; & R_{A}\left(u_{8}\right)=\left\{u_{8},u_{11},u_{12}\right\};\\ R_{A}\left(u_{9}\right)=\left\{u_{9},u_{10}\right\};\; & R_{A}\left(u_{10}\right)=\left\{u_{9},u_{10}\right\};\\ R_{A}\left(u_{11}\right)=\left\{u_{8},u_{11},u_{12}\right\};\; & R_{A}\left(u_{12}\right)=\left\{u_{8},u_{11},u_{12}\right\};\\ R_{A}\left(u_{13}\right)=\left\{u_{13}\right\};\; & R_{A}\left(u_{14}\right)=\left\{u_{14}\right\};\\ R_{A}\left(u_{15}\right)=\left\{u_{1},u_{2},u_{3},u_{15},u_{16},u_{17}\right\};\; & R_{A}\left(u_{16}\right)=\left\{u_{2},u_{15},u_{16},u_{17}\right\};\\ R_{A}\left(u_{17}\right)=\left\{u_{1},u_{2},u_{15},u_{16},u_{17},u_{18}\right\};\; & R_{A}\left(u_{18}\right)=\left\{u_{1},u_{2},u_{17},u_{18}\right\};\;\\ R_{A}\left(u_{19}\right)=\left\{u_{19},u_{20}\right\};\; & R_{A}\left(u_{20}\right)=\left\{u_{19},u_{20}\right\}. \end{array}$$

From Table A2, we can see that there are ten decision classes: $D_{1}=\left\{u_{1},u_{2}\right\}$, $D_{2}=\left\{u_{3},u_{4}\right\}$, $D_{3}=\left\{u_{5},u_{6}\right\}$, $D_{4}=\left\{u_{7},u_{8}\right\}$, $D_{5}=\left\{u_{9},u_{10}\right\}$, $D_{6}=\left\{u_{11},u_{12}\right\}$, $D_{7}=\left\{u_{13},u_{14}\right\}$, $D_{8}=\left\{u_{15},u_{16}\right\}$, $D_{9}=\left\{u_{17},u_{18}\right\}$, and $D_{10}=\left\{u_{19},u_{20}\right\}$. The lower approximation and upper approximation of each decision class $D_{l}$, l=1, 2, …, 10, were determined from Definition 5, as follows:

$$\begin{array}{ll} {\left(D_{1}\right)}_{A}=\phi ;\; & {\left(D_{1}\right)}^{A}=\left\{u_{1},u_{2},u_{15},u_{16},u_{17},u_{18}\right\}.\\ {\left(D_{2}\right)}_{A}=\left\{u_{4}\right\};\; & {\left(D_{2}\right)}^{A}=\left\{u_{3},u_{4},u_{15}\right\}.\\ {\left(D_{3}\right)}_{A}=\left\{u_{5},u_{6}\right\};\; & {\left(D_{3}\right)}^{A}=\left\{u_{5},u_{6}\right\}.\\ {\left(D_{4}\right)}_{A}=\left\{u_{7}\right\};\; & {\left(D_{4}\right)}^{A}=\left\{u_{7},u_{8},u_{11},u_{12}\right\}.\\ {\left(D_{5}\right)}_{A}=\left\{u_{9},u_{10}\right\};\; & {\left(D_{5}\right)}^{A}=\left\{u_{9},u_{10}\right\}.\\ {\left(D_{6}\right)}_{A}=\phi ;\; & {\left(D_{6}\right)}^{A}=\left\{u_{8},u_{11},u_{12}\right\}.\\ {\left(D_{7}\right)}_{A}=\left\{u_{13},u_{14}\right\};\; & {\left(D_{7}\right)}^{A}=\left\{u_{13},u_{14}\right\}.\\ {\left(D_{8}\right)}_{A}=\phi ;\; & {\left(D_{8}\right)}^{A}=\left\{u_{1},u_{2},u_{3},u_{15},u_{16},u_{17}\right\}.\\ {\left(D_{9}\right)}_{A}=\phi ;\; & {\left(D_{9}\right)}^{A}=\left\{u_{1},u_{2},u_{15},u_{16},u_{17},u_{18}\right\}.\\ {\left(D_{10}\right)}_{A}=\left\{u_{19},u_{20}\right\};\; & {\left(D_{10}\right)}^{A}=\left\{u_{19},u_{20}\right\}. \end{array}$$

The membership degrees of object u∈U belong to ${\left(D_{l}\right)}_{A}$ and ${\left(D_{l}\right)}^{A}$, l=1, 2, …, 10, and were calculated from Definition 6.

$$\begin{array}{l} \begin{array}{ll} \mu_{{\left(D_{1}\right)}_{A}}\left(u_{1}\right) & =\underset{v\in R_{A}\left(u_{1}\right)}{min}\left(max\left(1-R_{A}\left(u_{1},v\right),\hat{v}\right)\right)\\ & =min(max\left(1-R_{A}\left(u_{1},u_{1}\right),\;1\right),\;max\left(1-R_{A}\left(u_{1},u_{2}\right),\;1\right),\;\\ & max\left(1-R_{A}\left(u_{1},u_{15}\right),\;0\right);\;max\left(1-R_{A}\left(u_{1},u_{17}\right),\;0\right),\;max\left(1-R_{A}\left(u_{1},u_{18}\right),0\right)) \end{array}\\ =min\left(1.0,\;1.0,\;0.966,\;0.966,\;0.826\right)=0.826\\ \begin{array}{cc} \mu_{{\left(D_{1}\right)}^{A}}\left(u_{1}\right) & =\underset{v\in R_{A}\left(u_{1}\right)}{max}\left(min\left(R_{A}\left(u_{1},v\right),\hat{v}\right)\right)\\ & =max(min\left(R_{A}\left(u_{1},u_{1}\right),\;1\right),\;min\left(R_{A}\left(u_{1},u_{2}\right),\;1\right),\;min\left(R_{A}\left(u_{1},u_{15}\right),\;0\right),\\ & min\left(R_{A}\left(u_{1},u_{17}\right),\;0\right),\;min\left(R_{A}\left(u_{1},u_{18}\right),\;0\right))\\ & =max\left(1.0,\;0.274,\;0.0,\;0.0,\;0.0\right)=1.0. \end{array} \end{array}$$

The results of the membership degrees of each object belong to ${\left(D_{l}\right)}_{A}$ and ${\left(D_{l}\right)}^{A}$, as shown in Table A4.

**Table A4:** The membership degrees of each object belong to ${\left(D_{l}\right)}_{A}$ and ${\left(D_{l}\right)}^{A}$.

U	$\mu_{{\left(D_{1}\right)}_{A}}$	$\mu_{{\left(D_{1}\right)}^{A}}$	$\mu_{{\left(D_{2}\right)}_{A}}$	$\mu_{{\left(D_{2}\right)}^{A}}$	$\mu_{{\left(D_{3}\right)}_{A}}$	$\mu_{{\left(D_{3}\right)}^{A}}$	$\mu_{{\left(D_{4}\right)}_{A}}$	$\mu_{{\left(D_{4}\right)}^{A}}$	$\mu_{{\left(D_{5}\right)}_{A}}$	$\mu_{{\left(D_{5}\right)}^{A}}$	$\mu_{{\left(D_{6}\right)}_{A}}$	$\mu_{{\left(D_{6}\right)}^{A}}$	$\mu_{{\left(D_{7}\right)}_{A}}$	$\mu_{{\left(D_{7}\right)}^{A}}$	$\mu_{{\left(D_{8}\right)}_{A}}$	$\mu_{{\left(D_{8}\right)}^{A}}$	$\mu_{{\left(D_{9}\right)}_{A}}$	$\mu_{{\left(D_{9}\right)}^{A}}$	$\mu_{{\left(D_{10}\right)}_{A}}$	$\mu_{{\left(D_{10}\right)}^{A}}$
$u_{1}$	0.826	1.0	0	0	0	0	0	0	0	0	0	0	0	0	0	0.034	0	0.174	0	0
$u_{2}$	0.551	1.0	0	0	0	0	0	0	0	0	0	0	0	0	0	0.449	0	0.449	0	0
$u_{3}$	0	0	0.826	1.0	0	0	0	0	0	0	0	0	0	0	0	0.174	0	0	0	0
$u_{4}$	0	0	1.0	1.0	0	0	0	0	0	0	0	0	0	0	0	0	0	0	0	0
$u_{5}$	0	0	0	0	1.0	1.0	0	0	0	0	0	0	0	0	0	0	0	0	0	0
$u_{6}$	0	0	0	0	1.0	1.0	0	0	0	0	0	0	0	0	0	0	0	0	0	0
$u_{7}$	0	0	0	0	0	0	1.0	1.0	0	0	0	0	0	0	0	0	0	0	0	0
$u_{8}$	0	0	0	0	0	0	0.927	1.0	0	0	0	0.073	0	0	0	0	0	0	0	0
$u_{9}$	0	0	0	0	0	0	0	0	1.0	1.0	0	0	0	0	0	0	0	0	0	1.0
$u_{10}$	0	0	0	0	0	0	0	0	1.0	1.0	0	0	0	0	0	0	0	0	0	1.0
$u_{11}$	0	0	0	0	0	0	0	0.073	0	0	0.927	1.0	0	0	0	0	0	0	0	0
$u_{12}$	0	0	0	0	0	0	0	0.073	0	0	0.927	1.0	0	0	0	0	0	0	0	0
$u_{13}$	0	0	0	0	0	0	0	0	0	0	0	0	1.0	1.0	0	0	0	0	0	0
$u_{14}$	0	0	0	0	0	0	0	0	0	0	0	0	1.0	1.0	0	0	0	0	0	0
$u_{15}$	0	0.174	0	0.174	0	0	0	0	0	0	0	0	0	0	0.826	1.0	0	0.174	0	0
$u_{16}$	0	0.449	0	0	0	0	0	0	0	0	0	0	0	0	0.551	1.0	0	0.274	0	0
$u_{17}$	0	0.275	0	0	0	0	0	0	0	0	0	0	0	0	0	0.274	0.725	1.0	0	0
$u_{18}$	0	0.449	0	0	0	0	0	0	0	0	0	0	0	0	0	0	0.551	1.0	0	0
$u_{19}$	0	0	0	0	0	0	0	0	0	0	0	0	0	0	0	0	0	0	1.0	1.0
$u_{20}$	0	0	0	0	0	0	0	0	0	0	0	0	0	0	0	0	0	0	1.0	1.0

To build the fingerprinting decision rules database, we used Definition 7. Moreover, we used Definition 8 to calculate the credibility degree of the rule $\rho_{1}$ as follows:

$$\begin{array}{cc} \mu_{A}\left(\rho_{1}\right) & =\underset{u\in R_{A}\left(u_{1}\right)}{min}\left(max\left(1-R_{A}\left(u_{1},\;u\right),\mu_{D_{A}}\left(u\right)\right)\right)\\ & =min(max\left(1-R_{A}\left(u_{1},u_{1}\right),\mu_{{\left(D_{1}\right)}_{A}}\left(u_{1}\right)\right),\;max\left(1-R_{A}\left(u_{1},u_{2}\right),\;\mu_{{\left(D_{1}\right)}_{A}}\left(u_{2}\right)\right),\\ & \begin{array}{ll} & max\left(1-R_{A}\left(u_{1},u_{15}\right),\;\mu_{{\left(D_{8}\right)}_{A}}\left(u_{15}\right)\right),\;max\left(1-R_{A}\left(u_{1},u_{17}\right),\;\mu_{{\left(D_{9}\right)}_{A}}\left(u_{17}\right)\right),\\ & max\left(1-R_{A}\left(u_{1},u_{18}\right),\;\mu_{{\left(D_{9}\right)}_{A}}\left(u_{18}\right)\right)) \end{array}\\ & =min\left(0.826,\;0.726,\;0.966,\;0.966,\;0.826\right)=0.726. \end{array}$$

Furthermore, we used Definition 9 to determine the support object set of decision rule $\rho_{1}$, as follows:

$$S_{A}\left(\rho_{1}\right)=R_{A}\left(u_{1}\right)=\left\{u_{1},u_{2},u_{15},u_{17},u_{18}\right\}$$

The fingerprinting decision rules database is shown in Table A5.

**Table A5:** The fingerprinting decision rules database.

Decision Rule	$RSS_{1}\;(\mathrm{dBm})$	$RSS_{2}\;(\mathrm{dBm})$	$RSS_{3}\;(\mathrm{dBm})$	$RSS_{4}\;(\mathrm{dBm})$	$RSS_{5}\;(\mathrm{dBm})$	d	$\mathrm{Credibility}\;\mathrm{Degree}\;\left(\mu_{A}\left(\rho_{i}\right)\right)$	$\mathrm{Support}\;\mathrm{Object}\;\mathrm{Set}\;\left(S_{A}\left(\rho_{i}\right)\right)$
$\rho_{1}$	−57	−48	−64	−45	−52	$RP_{1}$	0.726	$\left\{u_{1},u_{2},u_{15},u_{17},u_{18}\right\}$
$\rho_{2}$	−58	−49	−63	−48	−53	$RP_{1}$	0.551	$\left\{u_{1},u_{2},u_{15},u_{16},u_{17},u_{18}\right\}$
$\rho_{3}$	−57	−43	−65	−51	−53	$RP_{2}$	0.826	$\left\{u_{3},u_{4},u_{15}\right\}$
$\rho_{4}$	−58	−42	−67	−51	−55	$RP_{2}$	0.826	$\left\{u_{3},u_{4}\right\}$
$\rho_{5}$	−55	−54	−63	−52	−57	$RP_{3}$	1.0	$\left\{u_{5},u_{6}\right\}$
$\rho_{6}$	−56	−51	−62	−52	−58	$RP_{3}$	1.0	$\left\{u_{5},u_{6}\right\}$
$\rho_{7}$	−40	−50	−57	−52	−53	$RP_{4}$	1.0	$\left\{u_{7}\right\}$
$\rho_{8}$	−43	−51	−61	−53	−53	$RP_{4}$	0.927	$\left\{u_{8},u_{11},u_{12}\right\}$
$\rho_{9}$	−41	−53	−62	−39	−50	$RP_{5}$	1.0	$\left\{u_{9},u_{10}\right\}$
$\rho_{10}$	−42	−53	−63	−39	−49	$RP_{5}$	1.0	$\left\{u_{9},u_{10}\right\}$
$\rho_{11}$	−50	−49	−61	−53	−50	$RP_{6}$	0.927	$\left\{u_{8},u_{11},u_{12}\right\}$
$\rho_{12}$	−50	−50	−61	−53	−51	$RP_{6}$	0.927	$\left\{u_{8},u_{11},u_{12}\right\}$
$\rho_{13}$	−50	−56	−65	−45	−48	$RP_{7}$	1.0	$\left\{u_{13}\right\}$
$\rho_{14}$	−51	−56	−69	−46	−49	$RP_{7}$	1.0	$\left\{u_{14}\right\}$
$\rho_{15}$	−57	−46	−65	−48	−56	$RP_{8}$	0.551	$\left\{u_{1},u_{2},u_{3},u_{15},u_{16},u_{17}\right\}$
$\rho_{16}$	−58	−47	−64	−50	−55	$RP_{8}$	0.551	$\left\{u_{2},u_{15},u_{16},u_{17}\right\}$
$\rho_{17}$	−59	−49	−63	−47	−56	$RP_{9}$	0.551	$\left\{u_{1},u_{2},u_{15},u_{16},u_{17},u_{18}\right\}$
$\rho_{18}$	−60	−51	−64	−47	−54	$RP_{9}$	0.551	$\left\{u_{1},u_{2},u_{17},u_{18}\right\}$
$\rho_{19}$	−67	−51	−66	−51	−42	$RP_{10}$	1.0	$\left\{u_{19},u_{20}\right\}$
$\rho_{20}$	−66	−53	−66	−51	−43	$RP_{10}$	1.0	$\left\{u_{19},u_{20}\right\}$

To test the positioning result in the online phase, we collected the RSS values at $RP_{1}$ with the set θ=(−56,−48,−62,−46,−51) (dBm), where each value is from one AP over five APs. Algorithm 2 of the VTRS–DR method was calculated, step by step, as follows.

Step 1: Calculate the valued tolerance relation between θ to the conditions of the rule $\rho_{i}$: $R_{A}\left(\theta ,\rho_{i}\right)$, i=1, 2,…, 20. The valued tolerance relation $R_{A}\left(\theta ,\rho_{i}\right)$ is greater than zero only for four rules, namely $\rho_{1}$, $\rho_{2}$, $\rho_{16}$, and $\rho_{18}$, with $R_{A}\left(\theta ,\rho_{1}\right)=0.213$, $R_{A}\left(\theta ,\rho_{2}\right)=0.516$, $R_{A}\left(\theta ,\rho_{16}\right)=0.032$, and $R_{A}\left(\theta ,\rho_{18}\right)=0.174$, respectively.

Step 2: The smallest value $\mu_{\rho_{i}}\left(\theta \right)$ between the valued tolerance relation $R_{A}\left(\theta ,\rho_{i}\right)$ and the credibility degree $\mu_{A}\left(\rho_{i}\right)$ of rule $\rho_{i}$ is greater than zero only for four rules, namely $\rho_{1}$, $\rho_{2}$, $\rho_{16}$, and $\rho_{18}$, with $\mu_{\rho_{1}}\left(\theta \right)=min\left(0.213,\;0.726\right)=0.213$, $\mu_{\rho_{2}}\left(\theta \right)=min\left(0.516,\;0.551\right)=0.516$, $\mu_{\rho_{16}}\left(\theta \right)=min\left(0.032,\;0.551\right)=0.032$, and $\mu_{\rho_{18}}\left(\theta \right)=min\left(0.174,\;0.551\right)=0.174$.

Step 3: The credibility degree $\mu_{D_{l}}\left(\theta \right)=\underset{\rho_{i}\in R\left(D_{l}\right)}{\max}\left(\mu_{\rho_{i}}\left(\theta \right)\right)$ is greater than zero only for three decision classes, namely $D_{1}$, $D_{8}$, and $D_{9}$, with $\mu_{D_{1}}\left(\theta \right)=\underset{\rho_{i}\in R\left(D_{1}\right)}{\max}\left(\mu_{\rho_{i}}\left(\theta \right)\right)=max\left(\mu_{\rho_{1}}\left(\theta \right),\mu_{\rho_{2}}\left(\theta \right)\right)$ =max(0.213, 0.516)=0.516, $\mu_{D_{8}}\left(\theta \right)=0.032$, and $\mu_{D_{9}}\left(\theta \right)=0.174$.

Step 4: Because $\exists !D_{1}\;:\;\mu_{D_{1}}\left(\theta \right)=0.516$ is the maximum, the new set θ belongs to the decision class $D_{1}$, meaning that Algorithm 2 returns the position of $RP_{1}$ (the right classified position); thus, the positioning error is 0 m.
